# Clinical and cost effectiveness of staff training in the delivery of Positive Behaviour Support (PBS) for adults with intellectual disabilities, autism spectrum disorder and challenging behaviour - randomised trial

**DOI:** 10.1186/s12888-020-02577-1

**Published:** 2020-04-15

**Authors:** Andre Strydom, Alessandro Bosco, Victoria Vickerstaff, Rachael Hunter, the PBS study group, Michaela Poppe, Victoria Ratti, Ian Hall, Jason Crabtree, Rumana Z. Omar, Asit Biswas, Jessica Blickwedel, Vivien Cooper, Mike Crawford, Angela Hassiotis

**Affiliations:** 1 Department of Forensic and Neurodevelopmental Sciences, Institute of Psychiatry Psychology and Neuroscience, King’s College London, 16 De Crespigny Park, London, SE5 8AF UK; 2 Division of Psychiatry, University College London, London, UK; 3 South London and Maudsley NHS Foundation Trust, London, UK; 4 Institute of Mental Health, University of Nottingham, Nottingham, UK; 5 Research Department of Primary Care and Population Health, University College London, London, UK; 6 Camden & Islington Foundation NHS Trust, London, UK

**Keywords:** Autism spectrum disorder, Intellectual disability, Challenging behaviour, Positive behaviour support

## Abstract

**Background:**

Although Positive Behaviour Support (PBS) is a widely used intervention for ameliorating challenging behaviour (CB), evidence for its use in adults with intellectual disability (ID) and comorbid autism (ASD) is lacking. We report a planned subsidiary analysis of adults with both ASD and ID who participated in a randomised trial of PBS delivered by health professionals.

**Methods:**

The study was a multicentre, cluster randomised trial conducted in 23 community ID services in England, participants were randomly allocated to either the delivery of PBS (*n* = 11 clusters) or to treatment as usual (TAU; *n* = 12). One-hundred and thirteen participants (46% of all participants in the trial) had a diagnosis of ID, autism spectrum disorder and CB (*ASD+*); (47 allocated to the intervention arm, and 66 to the control). CB (primary outcome) was measured with the Aberrant Behaviour Checklist total score (ABC-CT). Secondary outcomes included mental health status, psychotropic medication use, health and social care costs and quality adjusted life years (QALYs) over 12 months.

**Results:**

There were no statistically significant differences in ABC-CT between ASD+ groups randomised to the two arms over 12 months (adjusted mean difference = − 2.10, 95% CI: − 11.3 7.13, *p* = 0.655) or other measures. The mean incremental cost of the intervention per participant was £628 (95% CI -£1004 to £2013). There was a difference of 0.039 (95% CI − 0.028 to 0.103) for QALYs and a cost per QALY gained of £16,080.

**Conclusions:**

Results suggest lack of clinical effectiveness for PBS delivered by specialist ID clinical teams. Further evidence is needed from larger trials, and development of improved interventions.

**Trial registration:**

ClinicalTrials.gov: NCT01680276.

## Background

Autism Spectrum Disorder (ASD) is characterised by deficits in social communication and interaction, and restrictive and repetitive patterns of behaviour [1]. Current prevalence estimates report that ASD affects 52 million individuals worldwide [2, 3] and that there has been an increase in prevalence over the last two decades [4]. Many individuals with ASD also have intellectual disability (ID), which is characterised by significant impairment in intellectual and adaptive functioning [5]. Although estimates vary, ASD is a strongly associated with intellectual disability, with an estimated odds ratio of approximately 50 [6, 7]. Conversely, approximately 30% of individuals with ID are also diagnosed with ASD and individuals who have more severe ID are even more likely to have autism [8]. In the presence of a comorbid ASD and ID, more extensive care and support is usually needed as the dual diagnosis is associated with higher rates of restricted repetitive behaviours, and the domain of social functioning may be more affected in individuals with both ID and ASD compared to individuals with ID only [9, 10].

Challenging behaviour is defined as behaviour of such intensity, frequency or duration that the safety of the person and that of others is placed at risk and the access to ordinary community facilities is limited or denied [11]*.* ASD is an important predictor of challenging behaviour in addition to having more severe level of ID [12], but the lack of clear-cut diagnostic criteria to define challenging behaviour means there are marked variations in reported rates. For example, between 10% to more than 50% of individuals with ID and ASD may be reported to display various degrees of challenging behaviour [13–15].

Challenging behaviours in people with ID and comorbid ASD (*henceforth referred to as ASD+)* are more severe with a higher likelihood of long-standing presentation compared to individuals with ID only [1, 8, 16]. Self-injurious behaviour is 5 times more likely in ASD+ children and young people [17] and aggression may occur in 60% [18].

In addition, ASD+ individuals have an increased risk for comorbid mental illness (such as anxiety and sleep problems) compared to individuals with either ASD or ID only [19, 20]. For example, in a clinic sample from the National Health Service in England, 42% were diagnosed with other comorbid mental disorders including schizophrenia, depression, and anxiety [21]. These findings were replicated by Hove and Havik [22] who found that the presence of ASD in ID individuals was associated with higher levels of both mental disorders and challenging behaviour. In addition, symptoms of mental disorders appear to last longer in individuals with ASD+ [23] with consequently higher lifelong rates of depression and bipolar disorder [24]. Lunsky et al. [25] reported that both children and adults with ASD frequently attend hospital emergency departments in crisis, associated with exposure to family stress and/or negative life events. Presentation at psychiatry clinics and emergency departments may be particularly common in individuals with ASD+ [26].

Individuals with ASD are at increased risk of being socially excluded, and to receive poor quality care [27]. Costs of care for people with ASD are significant. In the UK, in 2011, ASD had an estimated impact on the UK economy of £32 billion [27]. In the USA, the total annual spending for ASD in 2015 was $268 billion, and it was predicted that it would rise to $461 billion in the following 10 years [28]. These costs comprise health and social care assessments and interventions, but there may be scope for more efficient use of resources by reducing preventable conditions such as challenging behaviour or improving use of evidence-based interventions. Another study by Knapp et al. [29] found increased use of respite care for adults with ASD+ compared to those with only ID. Therefore, there may be significant short and longer-term benefit in developing and testing effective interventions for people with ASD+; however, most of the efforts have focused on children and young people with only a few studies describing interventions targeting adults with ASD and ID [30]. Analysis of routinely collected data suggests that access to services may taper off as ASD+ children transition towards adulthood [31].

There are international calls for evidence-based psychosocial interventions in community settings to target both core ASD symptoms as well as associated comorbidities [32]. Few interventions for challenging behaviour have been tested using randomised controlled designs for efficacy. Positive Behaviour Support (PBS) is an intervention for challenging behaviour with wide appeal and used in many services across the UK and in other countries [33]. The defining features of PBS are a comprehensive therapeutic framework based on social, behavioural, educational, and medical stances combining mainly behavioural approaches such as Applied Behaviour Analysis (ABA), to improve the life of the individual and decrease challenging behaviours [34, 35]. PBS shares some commonalities with principles of operant psychology but includes a focus on cultural and social factors impinging on the onset and maintenance of challenging behaviour (e.g. educational settings, interpersonal dynamics, quality of life) [33, 36]. Whilst ABA is often delivered in special settings, e.g. school, and at home, it may be seen as potentially restrictive and has been criticised for not being person centred [37]. A multidimensional approach to the delivery of PBS comprises a series of assessments of staff support, physical and mental health morbidity, and organisational structures that may impact on the individual rather than aiming directly at the restructuring and modification of the challenging behaviour [33, 37]. A meta-analysis of small randomised and quasi-randomised clinical trials [38] suggested that behaviour interventions were effective in reducing challenging behaviour in the presence of autism, but these studies were small-scale or had methodological short-comings, and their clinical effectiveness in real-world settings remains uncertain.

The present analysis reports on the clinical and cost effectiveness of training health professionals working in community teams to deliver positive behaviour support for challenging behaviour in a subsample of adult ASD+ participants, testing a planned subsidiary hypothesis in a multicentre cluster randomised controlled trial in England [39, 40]. The trial was the first large-scale systematic evaluation examining the impact of the delivery of PBS by trained staff in secondary community health and social care ID services in the UK. The present objective was to carry out a planned analysis of the impact of the intervention and of the treatment as usual (TAU) alone at 12 months in a sub-sample of ASD+ participants, using the primary outcome measure (Aberrant Behaviour Checklist-Community total score).

## Methods

### Study design

The current study aims to investigate whether staff training in PBS delivery is clinically and cost effective compared to treatment as usual in adults with ASD+, in a trial described previously [40]. In summary, this was a multicentre, two-arm cluster randomised controlled trial in which 246 adults with ID (with and without ASD) were recruited from 23 community intellectual disability services across England.

### Sample size

The sample size for the main trial was calculated to detect a difference of 0.45 SD in the primary outcome (Aberrant Behaviour Checklist-Community total score) using 90% power and 5% significance level indicating that a minimum of 19 clusters and 246 participants were required. Recruitment of services and participants took place between June 2013 and January 2015. Ultimately, 11 services (*n* = 109 participants) were allocated to the intervention arm and 12 (*n* = 137) to TAU arm.

### Ethical approval, trial registration and consent

Ethical approval was issued by the NRES Committee London - Harrow (reference. 12/LO/1378). The trial was registered on ClinicalTrials.gov, identifier: NCT01680276.

Easy read information sheets and consent forms were used to obtain informed written consent from participants. Where a participant lacked capacity, a consultee acted on their behalf, in accordance with UK law.

### Clusters and participants

The 23 community intellectual disability services were randomly assigned to staff training in PBS and TAU or TAU alone. Participants were eligible to take part if they were at least 18 years old, had received a diagnosis of ID and displayed challenging behaviour (with an ABC-CT score of no less than 15 indicating a weekly experience of any type of challenging behaviour). Participants were excluded if they had a primary clinical diagnosis of personality disorder, substance misuse, relapse of a pre-existing mental disorder, or the clinical team decided that participation to the study would not be appropriate (e.g. participants with an acute illness episode or where there were significant issues around safeguarding of the individual at the time of the screening for inclusion in the study). Any services which had embedded PBS therapists or specialist behaviour teams were also excluded. Participants were assessed at baseline, and at 6 and 12 months post randomisation.

### Intervention

Therapists drawn from a variety of health professions, e.g. psychology, psychiatry, occupational therapy, speech and language therapy, received six-day manual assisted training in delivering PBS over 15 weeks. All professionals were employed by specialist community ID services and had experience in working with this patient group although some may have had limited experience of delivering behavioural interventions such as PBS. However, the training intervention was designed to be delivered by these health professionals rather than experienced behaviour specialists, in a real-world community setting.

In brief, the training was designed around the following topics:

- Functional Behavioural Assessment and formulation skills
- Primary Prevention
- Secondary Prevention and Reactive Strategies
- Periodic Service Review and Problem Solving.

All behavioural strategies taught via direct and didactic instruction are well established and depend on teaching the adult with ID alternative positive skills via his/her family or paid carers. The therapists were given a manual which described the rationale and treatment strategies and included printed and electronic materials and other resources, received individual feedback on their cases and associated paperwork, e.g. reports and formulations and received post-training mentoring by the trainers. They were further supported through monthly teleconferences with peers, the Chief Investigator, the trial manager and the trainers. Further details can be found in the full trial report [40].

### Treatment as usual (TAU)

In England, community lD teams provide comprehensive psychiatric and psychological supports to individuals with ID who present with challenging behaviour or mental health concerns. TAU comprised a variety of behavioural and/or psychosocial and pharmacological treatments as those being available in the services taking part in the study at that time [39].

### Allocation and blinding

An independent Web-based randomisation system (Sealed Envelope) was used, with random permuted blocks (1,1 allocation). Randomisation was stratified by the staff:patient ratio for each cluster using a binary factor indicating whether a cluster was below or above the median ratio. Whereas clusters, participants and carers were informed about group allocation, the Research Assistants (RAs) and Clinical Study Officers (CSOs) completing the study assessments were blind to treatment allocation. Where unblinding occurred, RAs from other locations who had remained blinded to arm allocation, carried out the study assessments.

### Measures

#### Primary outcome

Challenging behaviour was measured with the total Aberrant Behaviour Checklist-Community total score (ABC- CT) [41] at three assessment points, baseline, 6 and 12 months. The ABC-C consists of 5 dimensions of behaviour (Irritability, Agitation, Crying; Lethargy, Social Withdrawal; Stereotypic Behaviour; Hyperactivity, Noncompliance; Inappropriate Speech), each measured on a four-point rating scale (0–3). The total score is attained by summing up all the domain scores. The scale has very good psychometric properties and is widely used for monitoring of treatment effects [41].

#### Secondary outcomes

Mental health status of participants was measured with the Mini-Psychiatric Assessment Schedules for Adults with Developmental Disabilities (Mini PAS-ADD) [42]. This scale measures active mental health symptoms (over the past 4 weeks) and consists of 86 items that provide thresholds for 6 mental disorders: Depression, Anxiety, Obsessive Compulsive Disorder, Hypomania/Mania, Psychosis, Unspecified disorder. For the purpose of the analyses, those disorder categories were grouped into severe mental illness (SMI; psychosis and hypomania/mania) and into common mental disorders (CMD; depression, anxiety, obsessive-compulsive disorder).

Service use for the preceding 6 months based on patient or proxy responses was captured with the study adapted version of the Client Service Receipt Inventory (CSRI) [43]. Health related quality of life was measured by the EuroQol EQ-5D Youth version (EQ-5D-Y) to calculate Quality Adjusted Life Years (QALYs) in line with accepted guidance [44]. The EQ-5D-Y is a 5 dimension (usual activity, self-care, mobility, pain and anxiety/depression), 3 level (no problems, some problems and extreme problems) questionnaire. Family and paid carers completed it as proxies at baseline, 6 months and 12 months.

#### Other measures

Autism screen (carried out once at baseline): The Mini PAS-ADD includes 17 relevant questions which cover the following domains: impairments in social interaction (threshold = 4); impairments in communication (threshold = 1); repetitive stereotyped patterns of behaviour, interest and activities (threshold = 3). These items were used to describe the extent of autism symptoms. Participants’ level of functioning as proxy measure of intellectual disability was assessed with the short form of the Adaptive Behaviour Scale (SABS) [45] at baseline. Current medication prescriptions were also collected at each assessment point categorised into ‘any medications’ (i.e. for both physical and mental health), ‘antipsychotics’ and ‘other psychotropics’ (e.g. antidepressants, mood stabilisers).

#### Fidelity assessment

All documentation around treatment (i.e. PBS plan, goodness -of fit checklist, functional assessment and observational data) that was submitted by the therapists were assessed by an independent reviewer by means of the Behaviour Intervention Plan Quality Evaluation Scoring Guide II. The quality tool enabled the assessment and classification of the intervention plans as weak, underdeveloped, good or superior [46].

#### Ascertainment of ASD+ group

This group comprised all participants who were reported by paid or family carers to have had an established diagnosis of autism and which was recorded in the Case Report File.

**Fig. 1:**
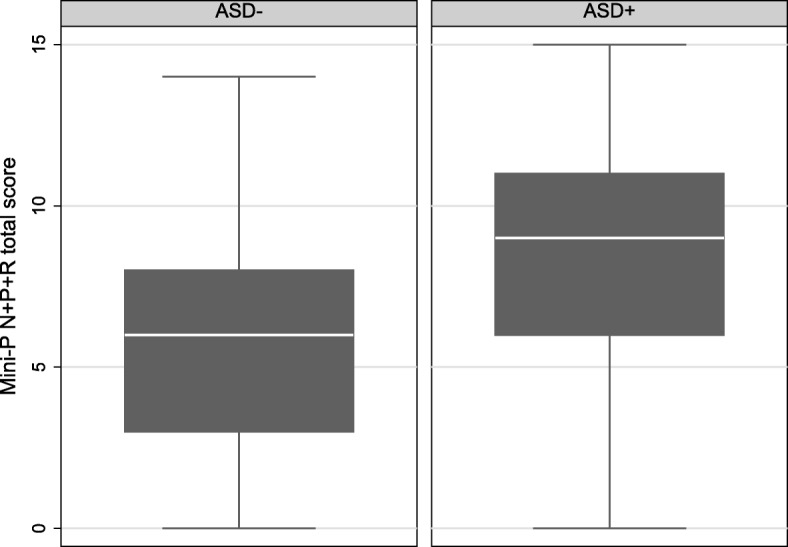
Comparison of Mini PAS-ADD scores b

This categorisation was further validated by comparing the Mini-PASADD autism subscale scores between the two diagnostic groups: ASD+ and ASD-; the ASD+ group scored significantly higher in all subscales as hypothesised (mean difference = 9.7 95%CI (2.5, 16.8); *p* = 0.008). The mean (SD) ABC- CT score in the ASD+ group was 70.7 (SD 29.5) compared to the ASD- group 61.0 (SD 27.1). The details are shown in Fig. 1.

### Statistical analysis

Before analysing the unblinded data, a statistical analysis plan was developed and agreed by the Trial Management Group, the Data Monitoring and Ethics Committee and by the Trial Steering Committee. All analyses were based on intention to treat. Differences in categorical and continuous variables according to ASD status were assessed using Chi-square, t-test or non-parametric equivalent. We used a three level random effects linear regression model adjusting for baseline ABC-CT measurements, the staff:patient ratio (low/high), time period and effects of clustering by services and repeated measures within subjects using random intercepts to examine the difference in the primary outcome, ABC-CT over 12 months for ASD+ participants randomized to the intervention compared to those randomised to TAU arms. The normality assumptions of the residuals were investigated using residual plots and were satisfied. A significance level of *p* < 0.05 was used. All analyses were by intention to treat using STATA version 14 [47].

### Analysis of service use and QALY

We calculated descriptive statistics for baseline, 6 and 12 months for community health and social care inputs, general medical and specialist mental health services use, comparing ASD+ participants in the control group versus those in the intervention group. Participants who reported using a service without specifically reporting the number of visits were included in the proportion using the service but could not be included in the calculation of means or standard deviation for number of visits. We provide total mean health and social care costs over 12 months for ASD+ participants allocated to the intervention and to TAU arms. Costs include the cost of the intervention.

Mean participant QALYs are calculated as the area under the curve adjusting for baseline imbalances [44].

Missing data for costs and QALYs was assumed to be missing at random and imputed using multiple imputations with chained equations. Variables identified as predictive of missingness across the trial were ID level, current living situation (alone or with others, and accommodation type (supported or independent) were included in the imputation model, with a percentage of missing data equal to 20%.

The mean incremental cost per QALY was calculated as the mean incremental cost of the intervention divided by mean incremental QALYs for the intervention. Mean incremental costs and QALYs were calculated using regression analysis, adjusting for baseline costs and staff to service user ratio and accounting for clustering by site as random effects. Ninety-five percent confidence intervals for health and social care costs and QALYs were calculated using bias corrected bootstrapping with 7000 draws using imputed data. The costing perspective is health and social care costs only and over 12 months. Units costs for care are reported in a previous publication by the authors [40]. Given the 12-month time horizon no discounting of costs and QALYs is included. All costs are in 2014/2015 British Pounds. A Cost-effectiveness plane of costs and QALYs for PBS training and delivery compared to TAU has been reported in the Additional file 1.

## Results

One hundred and thirteen (46.1%) participants were designated as ASD+ (47 participants in the intervention and 66 in the control arms). Demographic and clinical details of the ASD+ participants are shown in Table 1.

**Table 1:** Baseline ASD+ participants - demographics characteristics by intervention arm

N (%)	All ASD+ (*n* = 113)	TAU (*n* = 66)	Intervention (*n* = 47)	*P*-value
**Demographics**				
**Age**, years (mean, SD)	34.6 (14.0)	33.0 (14.0)	36.9 (14.0)	0.139
**Gender**, Male	83 (73)	89 (74)	34 (72)	0.821
**Ethnic origin**, White	75 (66)	44 (67)	31 (66)	0.937
**Short Form Adaptive Behaviour Scale (SABS)** (mean, SD)	46.9 (22.2)	46.6 (20.1)	47.2 (22.6)	0.900
**Current accommodation**				0.008
Residential	41 (36)	20 (30)	21 (45)	
Supported living	37 (33)	18 (27)	19 (40)	
Family home / Own house	35 (31)	28 (42)	7 (15)	
**Clinical**				
**Mini PAS-ADD** (n, %) (*n* = 241)				
Common mental disorder (CMD)	52 (47)	29 (45)	23 (50)	0.575
Severe mental illness (SMI)	20 (18)	11 (17)	9 (20)	0.681
**ABC (mean, SD)**				
Total score	70.7 (29.5)	74.4 (30.1)	65.5 (28.1)	0.114
Irritability	21.1 (11.1)	22.2 (10.9)	19.6 (11.2)	0.214
Lethargy	15.2 (9.2)	16.5 (9.9)	13.5 (7.9)	0.088
Stereotypy	7.4 (5.2)	7.6 (5.2)	7.1 (5.3)	0.654
Hyperactivity	21.9 (10.3)	23.1 (10.7)	20.3 (9.5)	0.144
Inappropriate speech	5.1 (4.3)	5.0 (4.4)	5.1 (4.3)	0.924
**Psychotropic medications**				
Any drug	102 (90)	60 (91)	42 (89)	0.7845
Antipsychotics	74 (65)	42 (64)	32 (68)	0.6239
**Physical health problems**				
Any physical health problem	78 (70)	48 (75)	30 (64)	0.203
Mobility^a^ (*n* = 78)	17 (22)	10 (21)	7 (23)	0.795
Sensory	15 (19)	12 (25)	3 (10)	0.102
Epilepsy	27 (35)	14 (29)	13 (43)	0.201
Incontinence	29 (37)	17 (35)	12 (40)	0.684
Other	49 (63)	32 (67)	17 (57)	0.374

^a^Of those with physical health problems, the number of people with the named problem. *ASD* Autism Spectrum Disorder, *ABC* Aberrant Behaviour Checklist, *ABS* Adaptive Behaviour Scale, *IQR* Interquartile range, *SD* Standard Deviation

### Intervention effect

#### Primary outcome

**Fig. 2:**
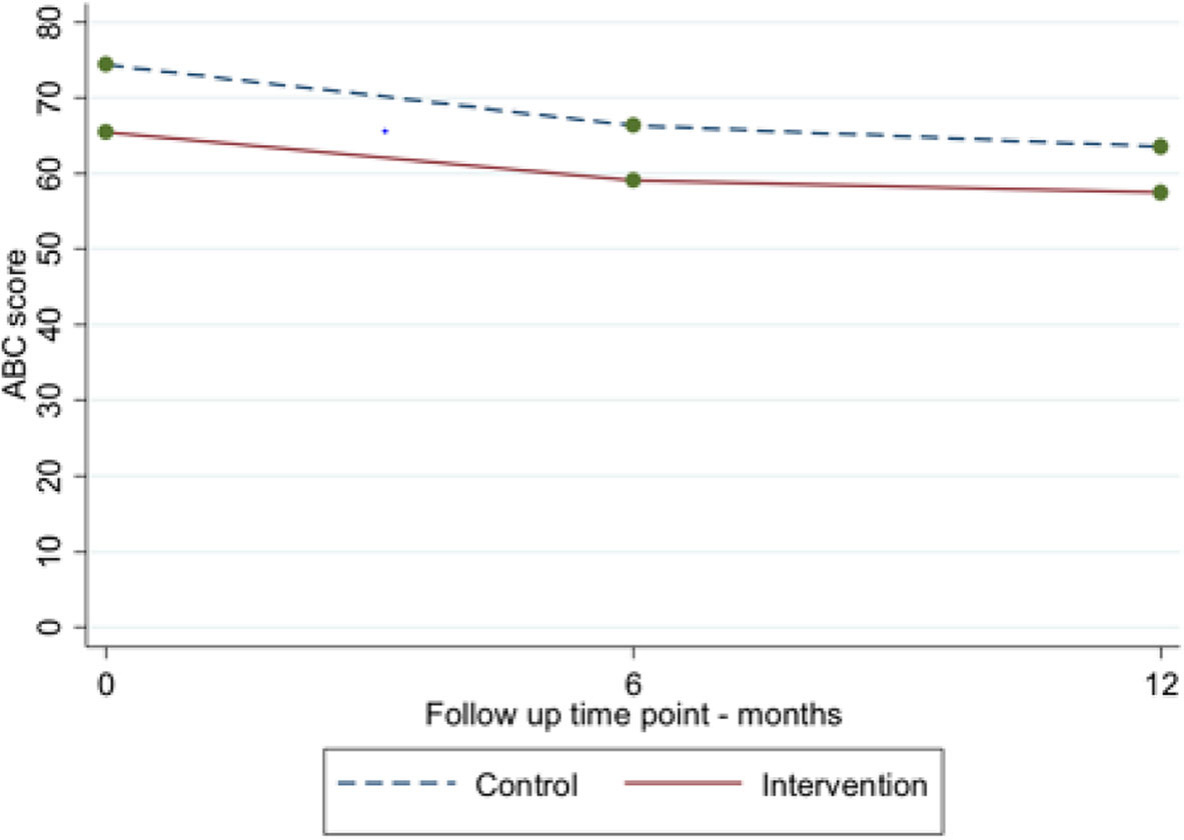
ABC-CT scores over 12 months for the ASD+ group

At baseline, the mean ABC-CT score in the intervention arm was 66 (SD 28) compared to 75 (SD 30) in the TAU arm (Additional file 2 for trial consort diagram). In the intervention arm, the mean ABC-CT reduced to 55 (SD 32) at 6 months and to 53 (SD 31) at 12 months. The respective mean scores in the TAU arm were 62 (SD 33) at 6 months and 60 (SD 29) at 12 months. The reduction in challenging behaviour over 12 months between the intervention and TAU arms (mean difference = − 2.10; 95% CI: − 11.3, 7.13; *p* = 0.655) was not statistically significant (Fig. 2). The intracluster correlation coefficient (ICC) for the ABC-CT score at the service level was 0.005 (95% CI: 0.000,1.000) and for the repeated measures within participants it was 0.579 (95% CI, 0.446, 0.701).

#### Secondary outcomes

There were no statistically significant differences between the ASD+ participants allocated to each trial arm in the ABC-C domain scores or in mental health status (see Table 2).

**Table 2:** ABC-C domain scores and mental health status over 12 months in ASD+ participants

ABC-C	Descriptive			Analysis over 12 months		
	Baseline	6 Months	12 Months	N_su_	Odds Ratio/Difference	95% CI
	Mean (SD)	Mean (SD)	Mean (SD)			
Total score						
TAU	74.4 (30.1)	66.4 (33.3)	63.6 (28.9)	107	−2.10	(−11.3, 7.13)
Intervention	65.5 (28.1)	59.2 (31.8)	57.5 (30.8)			
Irritability						
TAU	22.2 (10.9)	18.5 (10.7)	18.1 (10.6)			
Intervention	19.6 (11.2)	16.8 (11.2)	17.4 (10.7)	107	0.56	(−2.11, 3.22)
Lethargy						
TAU	16.5 (9.9)	16.6 (10.0)	15.4 (10.2)	107	−1.93	(−5.16, 1.29)
Intervention	13.5 (7.9)	13.2 (9.6)	12.4 (10.3)			
Stereotypy						
TAU	7.6 (5.2)	6.9 (5.7)	6.8 (5.0)	107	−0.36	(−1.74, 1.01)
Intervention	7.1 (5.3)	6.5 (5.8)	5.9 (5.7)			
Hyperactivity						
TAU	23.1 (10.7)	20.4 (12.0)	19.2 (10.0)	107	−0.02	(−3.16, 3.11)
Intervention	20.3 (9.5)	18.6 (11.5)	17.9 (10.8)			
Inappropriate speech				107	−0.014	(−1.31, 1.03)
TAU	5.0 (4.4)	4.2 (4.3)	4.0 (4.1)			
Intervention	5.1 (4.3)	4.1 (4.0)	4.0 (3.7)			
**Mini-PASADD**	**N (%)**	**N (%)**	**N (%)**			
Common mental disorder (CMD**)**						
TAU	29 (45)	23 (40)	26 (42)	105	1.00	(0.338, 2.97)
Intervention	23 (50)	20 (48)	16 (38)			
Severe mental illness (SMI)						
TAU	11 (17)	8 (14)	9 (15)	104	0.331	(0.023, 4.80)
Intervention	9 (20)	5 (12)	4 (10)			

*ASD* Autism Spectrum Disorder, *ABC-C* Aberrant Behaviour Checklist-Community, Mini *PAS-ADD* Psychopathology Assessment Schedule for Adults with Developmental Disabilities, *TAU* Treatment as usual

#### Medications

Overall, patterns of prescribing were similar in the ASD+ across the two study arms over the study duration. We noted that the proportion of individuals receiving antipsychotics in the intervention group increased slightly halfway through the trial, but proportions were similar by the end of the study. A slight reduction was observed in the use of other psychotropic medications over 12 months in the PBS arm, but at the end of the trial proportions were similar (details in Table 3).

**Table 3:** Psychotropic medication use over the trial duration in ASD+Participants

ASD+	Study arm	Baseline (PBS = 47; TAU = 66)	6 months (PBS = 42; TAU = 57)	12 months (PBS = 42; TAU = 63)
		**N (%)**		
**Any drug**	PBS	42 (89)	37 (88)	37 (88)
	TAU	60 (91)	51 (89)	57 (90)
**Antipsychotics**	PBS	32 (68)	31 (74)	27 (64)
	TAU	42 (64)	35 (61)	39 (62)
**Other psychotropic**	PBS	39 (83)	30 (71)	31 (74)
	TAU	41 (62)	41 (72)	47 (75)

#### Resource use

Descriptive statistics for community health and social care services, acute medical and specialist mental health services for ASD+ patients for the intervention and control arms are reported in Table 4. Means are reported only for those participants who have accessed the services.

**Table 4:** Service use by ASD+ at baseline, 6 and 12 months for the past 6 months

Publicly financed health care services		Baseline		6 months		12 months	
		Intervention	TAU	Intervention	TAU	Intervention	TAU
		N = 47	N = 66	*N* = 44	*N* = 62	N = 44	*N* = 63
**GP (any type of contact)**	N (%)^a^	43 (91%)	54 (82%)	36 (82%)	53 (85%)	37 (84%)	51 (81%)
	Mean^b^ (SD)	4.3 (3.9)	5.5 (8.8)	3.6 (3.6)	5.7 (6.9)	3.4 (3.4)	4.3 (4.6)
**Community nurse (District, ID)**	N (%)	25 (53%)	25 (38%)	20 (45%)	16 (26%)	18 (41%)	19 (30%)
	Mean (SD)	4.0 (4.9)	2.9 (2.6)	5.2 (6.1)	2.1 (1.6)	5.7 (9.4)	4.3 (7.1)
**Psychiatrist**	N (%)	34 (72%)	40 (61%)	29 (67%)t	37 (60%)	26 (59%)	38 (60%)
	Mean (SD)	1.6 (1.6)	1.7 (0.8)	1.4 (0.7)	2.0 (1.8)	1.3 (1.0)	2.1 (1.6)
**Other health professionals**	N (%)	17 (36%)	23 (35%)	13 (30%)	24 (39%)	19 (43%)	28 (44%)
	Mean (SD)	8.2 (19.1)	7.1 (9.8)	10.6 (19.6)	10.2 (18.9)	6.4 (11.6)	6.5 (8.0)
**Social Care including Community Support workers**	N (%)	31 (66%)	23 (35%)	24 (55%)	27 (44%)	24 (55%)	35 (56%)
	Mean (SD)	19.5 (54.4)	7.7 (26.8)	22.2 (56.0)	9.0 (17.8)	18.5 (51.7)	18.5 (46.1)
**Physiotherapy**	N (%)	3 (6%)	5 (8%)	1 (2.3%)	6 (10%)	3 (7%)	2 (3%)
	Mean (SD)	1 (0)	10.8 (9.7)	2	10.7 (11.6)	4.7 (6.4)	6.5 (7.8)
**Dentist**	N (%)	32 (68%)	38 (58%)	29 (67%)	37 (60%)	29 (66%)	34 (54%)
	Mean (SD)	1.5 (1.3)	1.5 (1.1)	1.6 (2.1)	1.9 (2.6)	1.4 (0.84)	1.4 (0.7)
***Acute and Specialist Services***							
**Inpatient/acute psychiatric ward**	N (%)	0	1 (2%)	0	0	0	1 (2%)
**Bed days**	Mean (SD)		21				188
***General Medical***							
**Admissions**	N (%)	1 (2%)	3 (5%)	0	0	2 (5%)	0
	Mean (SD)		1 (0)			2 (0)	
**Bed Days**	Mean (SD)	5	1.7 (1.2)			11.5 (13.4)	
***Medical ICU***^c^***/HDU***^d^							
**Admissions**	N (%)	1 (2%)	0	0	0	0	0
	Mean (SD)						
**Bed Days**	Mean (SD)	6					
**A&E Attendance (Physical Health)**	N (%)	7 (15%)	13 (20%)	4 (10%)	13 (21%)	5 (11%)	5 (8%)
	Mean (SD)	1.1 (0.9)	1.5 (1.8)	1 (0)	1.9 (2.5)	1.4 (0.5)	2 (1.7)
**A&E Attendance (Mental Health)**	N (%)	0	1 (2%)	0	0	1 (2%)	2 (3%)
	Mean (SD)						1.5 (0.7)
**Psychiatric outpatient**	N (%)	10 (21%)	16 (24%)	10 (24%)	21 (34%)	9 (21%)	15 (24%)
	Mean (SD)	1.5 (0.7)	1.6 (0.8)	1.2 (0.7)	1.9 (1.5)	2 (1.7)	1.5 (0.9)
**Day patient procedure**	N (%)	3 (6%)	15 (23%)	10 (24%)	18 (29%)	9 (21%)	15 (25%)
	Mean (SD)	1.3 (0.6)	1.5 (0.7)	1.4 (0.9)	1.4 (0.7)	1.4 (1.0)	1.8 (1.3)
**Medical outpatient**	N (%)	8 (17%)	15 (23%)	3 (7%)	11 (18%)	5 (11%)	7 (11%)
	Mean (SD)	2.9 (3.8)	1.7 (1.2)	4.3 (2.5)	3.1 (5.6)	2.3 (1.3)	6.4 (8.5)

^a^N = number of participants reporting use of the service in the past 6 months; ^b^: Mean contacts calculated for those that used the service; ^c^: intensive care unit (ICU); ^d^: high dependency unit (HDU); mean (SD) refers to the number of times individuals used a specific service

#### Incremental cost per QALY gained

Descriptive statistics for health and social care costs for ASD+ participants are reported in Table 5. The mean cost of health and social care service use over 12 months for ASD+ participants in the intervention arm is £2836 (SE 441) compared to £3433 (SE 770) in the control group. The mean incremental cost adjusted and calculated from imputed values and bootstrapping is -£969 (95% CI -£2603 to £415). The total cost of the intervention is £1598 per patient. When this is added to the total health and social care costs for intervention participants the mean incremental cost of the intervention compared to control is £628 (95% CI -£1004 to £2013). Descriptive statistics for EQ-5D-Y proxy tariff scores are reported in Table 6. The mean QALYs over 12 months for ASD+ participants in the intervention group is 0.623 (SE 0.039) compared to 0.546 (SE 0.035) with a mean incremental difference of 0.039 (95% CI − 0.028 to 0.103) based on imputed bootstrapped analysis. The mean incremental cost per QALY gained for the intervention compared to the control is £16,080 with a 63% probability that PBS is cost-effective at a £30,000 willingness to pay for a QALY gained (The probability was estimated from the cost-effectiveness acceptability curve using the proportion of bootstrap iterations where the net monetary benefit is greater than 0 when the willingness to pay for a QALY is £30,000). This is calculated for each bootstrap iteration using the net monetary benefit (NMB) formula of NMB = incremental benefit * willingness to pay – incremental cost). If the intervention were to cost more than £2140 per patient it would no longer be cost-effective at the £30,000 threshold.

**Table 5:** Mean health and social care costs per participant at 12 months for ASD+ participants

	Intervention (n = 44)	TAU (n = 62)
	Mean (SD)	
**Community costs including GPs, nursing, Allied Health and social care**	1199 (1449)	1403 (1731
**Mental health - secondary care**	65 (146)	781 (5341)
**Physical health - secondary care**	769 (1700)	823 (1292)
**Medication costs**	761 (1575)	436 (1215)

**Table 6:** EQ-5D-Y tariff scores at each time point – complete and imputed

EQ-5D Tariff scores		Baseline		6 months		12 months	
		Intervention	TAU	Intervention	TAU	Intervention	TAU
EQ-5D-Y Proxy responses	N	*N* = 46	N = 63	*N* = 43	*N* = 55	N = 43	N = 62
	Mean (SD)	0.585 (0.356)	0.508 (0.353)	0.627 (0.324)	0.583 (0.321)	0.638 (0.301)	0.522 (0.305)
EQ-5D-Y Proxy imputed	Mean (SE)			0.629 (0.049)	0.566 (0.042)	0.631 (0.046)	0.526 (0.040)

*SD* Standard deviation, *SE* Standard error, *TAU* Treatment as usua

## Discussion

The present analysis using data from a cluster randomised trial of staff training in delivering PBS suggests that the intervention did not reduce challenging behaviour in ASD+ participants.

These findings are in keeping with the main trial findings, which showed no effect of staff training in PBS on reducing challenging behaviour [40]. In line with other studies [48–52], ASD+ participants displayed a high level of challenging behaviour. Over 60% of ASD+ participants were receiving antipsychotics or other psychotropic medication for the duration of the study [53]. However, the proportion of individuals receiving such medication, especially antipsychotics, fluctuated over the study period and appeared to be initially increased in the intervention arm, but later the proportions dropped back to a similar level with those in the control arm at the end of the trial.

The proportion of ASD+ participants with comorbid common mental disorders at around 45% is in line with the prevalence reported in a meta-analysis by Hollocks et al. [54]. About 15% appeared to suffer with severe mental illness which is lower than the previously reported prevalence of up to 28% [55].

Whilst there were several admissions and emergency attendances for physical ill health, only one participant was admitted to an inpatient psychiatric unit during the follow up period, with another two having a mental health crisis. In contrast, Tsakanikos et al. [21], using routinely collected data from NHS mental health services, found that ASD contributed to psychiatric inpatient admissions in adults with ID. In our study, a quarter of ASD+ participants were seen by psychiatrists and a third were in contact with other health professionals, e.g. psychologists, which is arguably low given the significant mental health comorbidity in this sample. Access to services may be influenced by both perceived but also by unrecognised need.

Therefore, our findings may also reflect the existing gap of services for ASD+ individuals who have expressed concerns about the lack of ‘autism-specific services’ and the consequent inability to have their needs met by existing service provision [56]. Other factors, such as age of participants may also play a role, given that a study using Medicaid records suggested that aging with ASD tends to increase use of hospital-based services but that demand for community-based services gradually declines [57]. Whilst the US and UK health systems are vastly different, such trends will have implications for service planning and consequent impact on functional outcomes. Counterintuitively, despite the lack of effect on the main outcome measure, the intervention was cost-effective based upon QALYs for ASD+ participants at a £30,000 cost-effectiveness threshold used by the National Institute for Health and Care Excellence.

### Strength and limitations

To our knowledge, this study is the first to explore the effectiveness of staff training to deliver PBS to ASD+ adult participants with challenging behaviour in *real world* conditions. Most other studies of effectiveness of psychosocial interventions are conducted with child populations, include few individuals with intellectual disabilities and are often carried out in specialised settings [12]. The sample is representative of people with intellectual and developmental disabilities registered with community services within the National Health Service (NHS) in England [58]. The diagnosis of ASD used both screening and clinical information as recorded in the study Case Report File and was validated. Our approach to identifying ASD+ adults is justified by previous research which suggested that ASD+ young adults have higher rates of “episodic psychiatric disorder” than those without ASD [24]. Another strength is the varied professions of the therapists, which represents the range of expertise found in the community ID teams in the UK. However, as intellectual disability teams in the UK already provide comprehensive and expert services to individuals with ID and ASD who present with challenging behaviour, we accept that clinical effectiveness may have been reduced by similarities in the intervention between the intervention and TAU arms. Against this, we argue that the TAU arm likely did not include evidence-based interventions due to the general lack of evidence in the field.

Another factor that may have contributed to the intervention failing to show a difference may be low treatment fidelity [40], which indicated that about a third of participants in the intervention arm have had all the elements of the intervention implemented (observations, drawing up of a PBS plan and review of the plan to monitor implementation). This reflects the realities of a “real-world” setting, suggesting that adaptations may be required to improve the implementation of complex interventions such as PBS. The effect of training in interventions also tends to dissipate with time and this may have also occurred in the trial despite efforts to maintain mentoring and supervision throughout [59].

Whilst a number of psychosocial approaches are being developed and tested for individuals with ASD with or without ID in childhood [60], there is a relative lack of such approaches in adulthood particularly for a disorder with significant genetic burden [61]. Therefore, whilst it is possible that the delivery of PBS was less than optimal, there is also a question as to whether such behavioural approaches may need to be adapted to meet the needs of adults and their families.

A significant proportion of people with intellectual disabilities accessing community services also have ASD, and PBS is often the only evidence based psychological approach available for the treatment of challenging behaviour. Guidelines [12] suggest that challenging behaviour in the absence of a comorbid mental or physical condition should include a functional analysis with “clearly defined intervention strategies”. Staff training in PBS places emphasis on the function of the behaviour, e.g. escaping from demands, gaining attention, using Applied Behaviour Analysis [62]. However, about a third of functional assessments fail to identify the reasons behind the behaviour, or it may be refractory to the removal of the trigger or by shaping the function [63].

Finally, the sample size calculation of the original study was based on a pilot trial [64] which may have overestimated the effect of training in PBS as delivered by a specialist team in one area in England. There is not as yet any work available that has identified the Minimum Clinically Significant Difference (MCID) for the primary outcome used in this study; although MCID is not a new concept in health care research, it has yet to be integrated in the field of ID where the number of efficacy and effectiveness trials are still limited [65]. In addition, as carers and family members who were not blinded to treatment allocation reported the EQ-5D measure, this may have introduced bias in these scores.

## Conclusions

This analysis shows that PBS implemented via training of specialist health care staff in ID teams working in real-world settings with a broad range of patients with ID with ASD and challenging behaviour may be less effective than suggested in small-scale earlier studies of highly selected cases. Day to day care for ASD+ individuals requires refinement of interventions that can be delivered by a wide range of professionals. Improving implementation of PBS via adaptation of the intervention to take account of issues such as size and heterogeneity of caseloads, and identifying response markers to target it better, will be paramount to its feasibility and fidelity. Future studies are needed to examine the longer-term effectiveness of training in PBS for ASD+ using a variety of methodologies including primary data from clinical trials or other available datasets, and to develop and test new interventions.

## Supplementary information

**Additional file 1.** Cost-effectiveness plane of costs and QALYs for PBS training and delivery compared to TAU from a health care cost perspective over 12 months

**Additional file 2.** Trial Consort Diagram (12 months). Adapted from Hassiotis et al. (2018). Reporting of study flow diagram per ASD diagnosis.

## Data Availability

The anonymised dataset supporting the conclusions of this article is available in the UCL repository, (http://discovery.ucl.ac.uk). (10.14324/000.ds.10041531).

## Abbreviations

ABC-C: Aberrant behavior checklist-community
ASD: Autism spectrum disorder
ASD +: Participants with both ID and ASD
CMD: Common mental disorders
ID: Intellectual disability
PBS: Positive behaviour support
QALY: Quality adjusted life years
SMI: Severe mental illness
TAU: Treatment as usual
