# Supplementary material for: Clinical and cost effectiveness of staff training in the delivery of Positive Behaviour Support (PBS) for adults with intellectual disabilities, autism spectrum disorder and challenging behaviour - randomised trial
Source: BMC Psychiatry. 2020 Apr 15;20:161. doi: 10.1186/s12888-020-02577-1 (PMC7158144; doi:10.1186/s12888-020-02577-1)
Supplement: Supplementary file 1 — Additional file 1. Cost-effectiveness plane of costs and QALYs for PBS training and delivery compared to TAU from a health care cost perspective over 12 months [file 12888_2020_2577_MOESM1_ESM.docx]

**Additional file 1: Cost-effectiveness plane of costs and QALYs for PBS training and delivery compared to TAU from a health care cost perspective over 12 months**

**
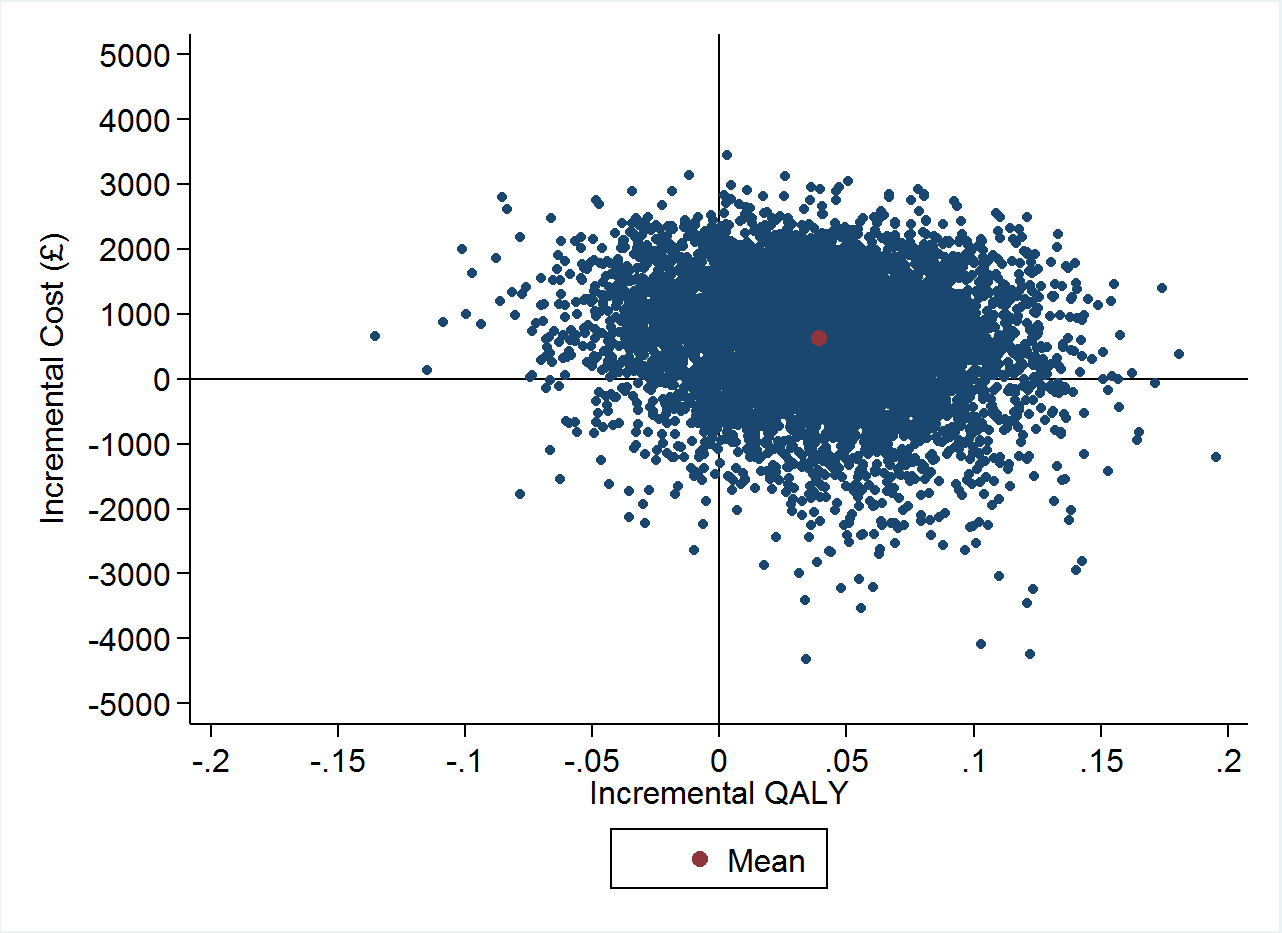
**
