# Supplementary material for: Clinical and cost effectiveness of staff training in the delivery of Positive Behaviour Support (PBS) for adults with intellectual disabilities, autism spectrum disorder and challenging behaviour - randomised trial
Source: BMC Psychiatry. 2020 Apr 15;20:161. doi: 10.1186/s12888-020-02577-1 (PMC7158144; doi:10.1186/s12888-020-02577-1)
Supplement: Supplementary file 2 — Additional file 2. Trial Consort Diagram (12 months). Adapted from Hassiotis et al. (2018). Reporting of study flow diagram per ASD diagnosis. [file 12888_2020_2577_MOESM2_ESM.docx]

Additional file 2. Trial Consort Diagram (12 months). Adapted from Hassiotis et al. (2018).

**Teams Excluded**

Refuse to take part

N_t_ = 5

**Service User Excluded**

Ineligible N_su_=1

**Service User Excluded,** N_su_= 136

Reached recruitment target: 52,

No consent provided: 42,

Ineligible: 22,

Moved out of area: 7,

Adverse event: 5,

Uncontactable: 4,

Not screened in time: 4

**Service users recruited,**

N_su_= 246; N_ASD+_= 113

**Number of teams**, N_t_= 23

**Service users screened**, N_su_= 382

**Number of teams recruited**

N_t_ = 28

**Allocated to Intervention**

N_t_=11

N_su_=108

N_ASD+_= 47

N_pc=_ ; N_fc=_

Deliver PBS training

**Allocated to TAU**

N_t_=12

N_su_=137

N_ASD+_= 66

**Time 2 (6 months) assessments**

N_su=_98

N_ASD+_= 44

**Time 2 (6 months) assessments**

N_su_=117

N_ASD+_= 62

**Time 3 (12 months) assessments**

N_su_= 100

N_ASD+_= 44

**Time 3 (12 months) assessments**

N_su_= 125

N_ASD+_= 63

**Abbreviations**

N_t_ Number of teams N_su_ Number of service users

N_ASD+_ Number of service users with ID and ASD included in this analysis.
